# Photon-Counting Detector CT Scan of Dinosaur Fossils: Initial Experience

**DOI:** 10.3390/jimaging11060180

**Published:** 2025-06-02

**Authors:** Tasuku Wakabayashi, Kenji Takata, Soichiro Kawabe, Masato Shimada, Takeshi Mugitani, Takuya Yachida, Rikiya Maruyama, Satomi Kanai, Kiyotaka Takeuchi, Tomohiro Kotsuji, Toshiki Tateishi, Hideki Hyodoh, Tetsuya Tsujikawa

**Affiliations:** 1 Department of Radiology, Faculty of Medical Sciences, University of Fukui, 23-3 Matsuoka-Shimoaizuki, Eiheiji 910-1193, Fukui, Japan; task@u-fukui.ac.jp (T.W.); tkenji@u-fukui.ac.jp (K.T.); satomi-k@u-fukui.ac.jp (S.K.); kiyo3266@u-fukui.ac.jp (K.T.); tkotsuji@u-fukui.ac.jp (T.K.); 2 Faculty of Dinosaur Paleontology, Fukui Prefectural University, 4-1-1 Matsuoka Kenjojima, Eiheiji 910-1195, Fukui, Japan; kawabe_soichiro@yahoo.co.jp; 3 Fukui Prefectural Dinosaur Museum, 51-11 Terao, Muroko, Katsuyama 911-8601, Fukui, Japan; 4 Radiological Center, University of Fukui Hospital, 23-3 Matsuoka-Shimoaizuki, Eiheiji 910-1193, Fukui, Japan; mshima@u-fukui.ac.jp (M.S.); ta1125@g.u-fukui.ac.jp (T.M.); yachida@g.u-fukui.ac.jp (T.Y.); rikiyam@u-fukui.ac.jp (R.M.); ttateish@g.u-fukui.ac.jp (T.T.); 5 Department of Forensic Medicine, Faculty of Medical Sciences, University of Fukui, 23-3 Matsuoka-Shimoaizuki, Eiheiji 910-1193, Fukui, Japan; hyodoh@u-fukui.ac.jp

**Keywords:** PCT-CT, paleontology, dinosaur fossil

## Abstract

Beyond clinical areas, photon-counting detector (PCD) CT is innovatively applied to study paleontological specimens. This study presents a preliminary investigation into the application of PCD-CT for imaging large dinosaur fossils, comparing it with standard energy-integrating detector (EID) CT. The left dentary of *Tyrannosaurus* and the skull of *Camarasaurus* were imaged using PCD-CT in ultra-high-resolution mode and EID-CT. The PCD-CT and EID-CT image quality of the dinosaurs were visually assessed. Compared with EID-CT, PCD-CT yielded higher-resolution anatomical images free of image deterioration, achieving a better definition of the *Tyrannosaurus* mandibular canal and the three semicircular canals of *Camarasaurus*. PCD-CT clearly depicts the internal structure and morphology of large dinosaur fossils without damaging them and also provides spectral information, thus allowing researchers to gain insights into fossil mineral composition and the preservation state in the future.

## 1. Introduction

Photon-counting detector (PCD) computed tomography (CT) is a next-generation imaging technology capable of measuring individual X-ray photon energy that is converted into an electrical signal [1,2]. PCD-CT has many advantages over conventional energy-integrating detector CT (EID-CT) such as high spatial resolution, low electronic noise, improved contrast-to-noise ratio, and spectral information acquisition. There are many clinical applications of PCD-CT, from the detection of minute calcifications in cardiovascular disease to precise cancer tumor mapping [3].

Beyond its clinical applications, CT is an established and valuable tool in paleontology for studying specimens such as dinosaur fossils, with these investigations increasingly benefiting from deep learning algorithms [4,5,6,7]. This non-destructive imaging technique enables internal fossil examination without requiring physical sectioning, thus preserving the integrity of these invaluable specimens. By leveraging PCD-CT’s high-resolution capabilities, researchers will gain insights into the internal morphology and pathology of fossils, shedding light on the life and environment of ancient organisms.

This paper reports our initial experience using PCD-CT in ultra-high-resolution (UHR) mode for dinosaur fossil imaging, highlighting the synergistic benefits of this technology for both medical and paleontological research. Through an interdisciplinary approach, we illustrate PCD-CT’s potential for new discoveries in the field of paleontology.

## 2. Materials and Methods

### 2.1. Dinosaur Fossils

This study was approved by the Ethics Committee of the University of Fukui, Faculty of Medical Sciences (study protocol # 20220056, 29 June 2023). Two large dinosaur fossils (the left dentary of *Tyrannosaurus rex* (FPDM-V-9767) and the skull of *Camarasaurus* sp. (FPDM-V-8509)) stored in the Fukui Prefectural Dinosaur Museum (Katsuyama City, Fukui, Japan) were carefully transported to the University of Fukui Hospital. 

The carnivorous *Tyrannosaurus rex* lived during the late Cretaceous period, around 68 to 66 million years ago [8,9]. It is one of the most well-known dinosaurs, recognized for its large size and fearsome appearance. Adult *T. rex* specimens are recorded at lengths of up to 40 feet (12 m) and weigh up to 9 tons. The *T. rex* had a massive body, strong legs, and powerful jaws with sharp teeth. Studies of *T. rex*’s teeth and jaws suggest that it possessed a crushing bite force that indicates a diet likely including both flesh and bone. Paleontologists believe it was an apex predator, preying upon herbivorous dinosaurs and scavenging as well.

*Camarasaurus* is a sauropod (long-necked) dinosaur that lived during the Late Jurassic period, around 155 to 145 million years ago [10,11]. This dinosaur is recognized for its large size and is the most common North American sauropod fossil. Adult *Camarasaurus* specimens are recorded at lengths of up to 50 feet (15 m) with estimated weights of up to 20 tons. *Camarasaurus* had a robust build, with a distinctive small, square head balanced by a long, heavy tail. Paleontologists believe that it fed on plant material by the shape and wear patterns of its teeth.

### 2.2. CT Systems

The first clinical PCD-CT system (NAEOTOM Alpha, Siemens Healthineers, Forchheim, Germany) with a large 82 cm aperture uses a dual-source geometry and 0.25-second gantry rotation time to provide 66 msec temporal resolution (isocenter) [12,13,14]. The two detector arrays consist of dedicated PCDs with 1.6 mm thick CdTe and have 50 cm and 36 cm scanning field of views, respectively. The detector pixel layout shows 0.151 × 0.176 mm^2^ subpixels (measured at the isocenter). Due to the presence of collimator blades every 6 subpixels in the z-direction, the minimum effective section thickness is 0.2 mm in UHR mode and 0.4 mm in standard mode. The UHR mode features 120 detector rows (120 × 0.2 mm collimation), while the standard mode features 144 detector rows (144 × 0.4 mm collimation).

The Aquilion Lightning Helios i Edition, developed by Canon Medical Systems (Otawara, Tochigi, Japan), is an 80-slice/160-slice EID-CT scanner with a 78 cm aperture and enables a high-speed helical scan of 0.5 mm × 80 slices.

## 3. Results

**Figure 1:**
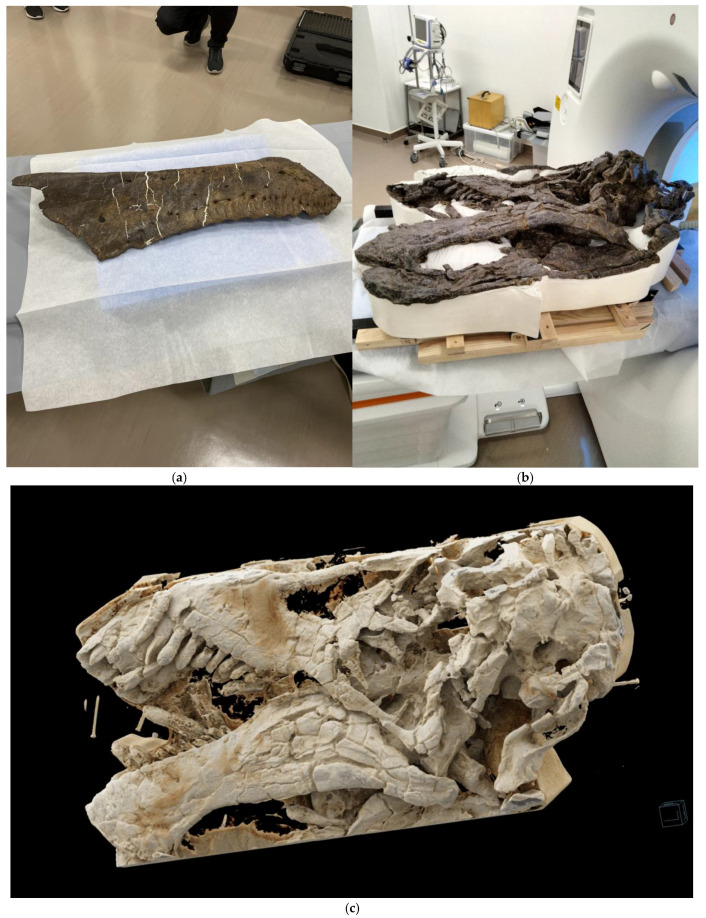
A part of the left dentary of *Tyrannosaurus* (**a**) and the entire skull of *Camarasaurus* (**b**) placed on the PCD-CT bed; a 3D image of the entire skull of *Camarasaurus* taken with PCD-CT (**c**).

The weights and dimensions of the dinosaur fossils are listed in Table 1. Imaging and reconstruction parameters for PCD-CT and EID-CT are listed in Table 2. The large 82 cm gantry bore of the PCD-CT was large enough to photograph part of the left dentary bone of *Tyrannosaurus* and the entire skull of *Camarasaurus* (Figure 1a,b, respectively). Figure 1c shows a 3D image of the entire *Camarasaurus* skull taken using PCD-CT.

**Figure 2:**
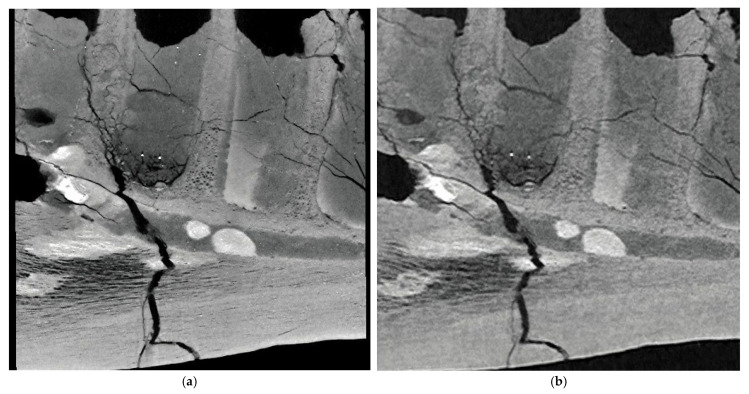
Sagittal sections of the *Tyrannosaurus* dentary bone imaged with PCD-CT (**a**) and EID-CT (**b**).

Figure 2a,b show the sagittal sections of the *Tyrannosaurus* dentary imaged with PCD-CT and EID-CT, respectively. Compared with EID-CT, PCD-CT more clearly depicts the cross-sectional patterns and the mandibular canal including a hyper-dense structure due to its apparent lower noise.

**Figure 3:**
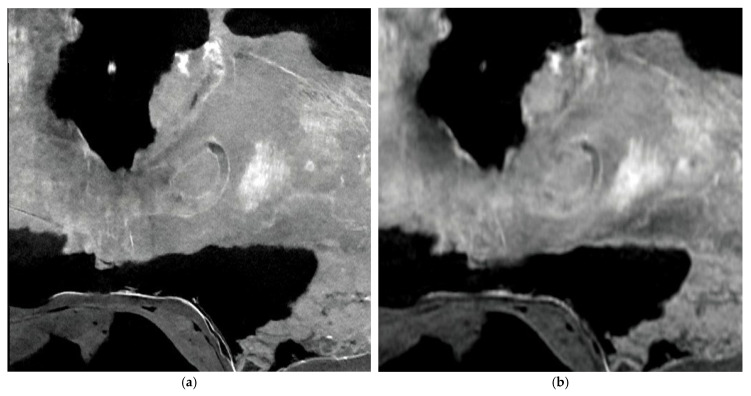
Cross-sections of the *Camarasaurus* temporal bone imaged with PCD-CT (**a**) and EID-CT (**b**).

Figure 3a,b show the cross-sections of the *Camarasaurus* temporal bone imaged with PCD-CT and EID-CT, respectively. The EID-CT image appears more blurred compared to PCD-CT, and PCD-CT more clearly depicts the selected section of the semicircular canals.

## 4. Discussion

To the best of our knowledge, this is the first application of PCD-CT in dinosaur fossil imaging. This study demonstrates the significant advantages of PCD-CT over conventional EID-CT in imaging large dinosaur fossils. The superior spatial resolution and contrast-to-noise ratio of PCD-CT allow for a more detailed visualization of fossil internal structures such as the *Tyrannosaurus* mandibular canal and the semicircular canals of *Camarasaurus*. For Figure 2, we explicitly note the apparent lower noise in the PCD-CT image compared to the EID-CT image. Regarding the apparent lower noise, the CTDI and DLP were higher in the PCD-CT than in the EID-CT (Table 2), which can help reducing noise in every detector configuration. In addition, this could be attributed to the inherent characteristics of PCDs, such as the elimination of electronic noise and efficient energy weighting. For Figure 3, we acknowledge that the EID-CT image appears more blurred compared to the PCD-CT image. One of the potential contributing factors is the possibility of increased scatter radiation effects in EID-CT systems when imaging dense and large objects like fossils. The energy discrimination capabilities of PCD-CT might help mitigate such effects, leading to improved image sharpness. Visualizing fine anatomical details with PCD-CT creates new possibilities for paleontological research. For example, detailed images of the mandibular canal in the *Tyrannosaurus* fossil specimen may provide new information on the sensory capabilities and feeding behavior of this iconic dinosaur [15]. Similarly, the clear imaging of the semicircular canals in the Camarasaurus fossil specimen may shed light on the balance and locomotion of this large sauropod.

Non-destructive PCD-CT imaging ensures that valuable fossils are studied in detail without the risk of damage. One additional benefit of PCD-CT is its ability to create effective atomic number images from spectral information, which may be used for future analysis of fossil composition (i.e., the weathering phenomenon known as pyrite disease that affects fossils). Pyrite disease occurs when fossils are extracted from rock and placed in oxygen-rich environments or exposed to rising temperatures, which accelerate oxidation [16]. As a result, the crystals of pyrite or marcasite (FeS_2_) grow within the fossil, causing it to deteriorate from the inside. Pyrite disease is a significant concern for the preservation of specimens, as it often affects dinosaur fossils. However, the progression of pyrite disease within fossils and the ultimate destruction it causes have not been thoroughly investigated. The ability of PCD-CT to create effective atomic number images from spectral information provides great potential for non-destructive testing and analysis in paleontology, allowing researchers to gain insights into fossils’ mineral composition and preservation state without damaging them.

This preliminary study has some limitations, especially from the quantitative point of view, for PCD-CT in paleontology. First of all, paleontologists using CT instrumentation are mainly interested in quantitative morphometry, implying some sort of image segmentation [6,7]. The quality of segmentation depends not only on numerous image quality parameters, such as spatial resolution, but also on image uniformity, low noise, and the absence of artifacts (such as, for instance, beam hardening). With such a thick bony structure, it could be highlighted how PCD-CT behaves in terms of beam hardening artifacts and HU level uniformity in homogeneous areas (e.g., far from metallic contaminants), in comparison to EID-CT. Furthermore, the choice of the virtual monochromatic energy (e.g., 70 keV) could have an impact on the image quality and discrimination of contaminants. Importantly, it shall be more emphasized how reliable a simple threshold-based segmentation could be on the virtual monochromatic image (VMI) from PCD-CT with respect to the same segmentation approach on the EID-based image. Further investigation is warranted, including a more in-depth analysis of beam hardening artifacts and HU level uniformity, the impact of virtual monochromatic energy choices, and quantitative comparisons of segmentation results, volume/surface measurements, and HU value distributions, among others.

Deep learning reconstruction (DLR) represents a state-of-the-art technique for enhancing image quality in classical EID-CT, including its application in fossil imaging. However, for our current study focusing on the initial application of PCD-CT, a direct comparison with DLR specific to our PCD-CT system is not yet feasible. PCD-CT is a very new technology, and as such, the extensive and specialized datasets required to train DLR algorithms for this particular modality (Siemens NAEOTOM Alpha, Forchheim, Germany) and for such unique applications as large fossil imaging are not yet established or available. The development of DLR for PCD-CT is an ongoing process that will require substantial data acquisition and research. As PCD-CT technology matures and more data become available, future work could indeed explore the potential synergies of DLR with PCD-CT data to further enhance image quality in fossil studies.

The high cost and limited availability of PCD-CT scanners may restrict their use to well-funded research institutions. Additionally, PCD-CT image interpretation requires specialized knowledge and expertise, which may not be readily available in all paleontological research settings. Thus, it is important for radiologists and paleontologists to collaborate and complement each other’s knowledge to advance PCD-CT research on dinosaur fossils.

## 5. Conclusions

This study highlights PCD-CT as a powerful tool for the non-destructive imaging of large dinosaur fossils. The superior image quality and additional spectral information provided by PCD-CT enhance our understanding of the fossil internal structure and composition and pave the way for new discoveries in paleontology. Future research should focus on expanding the use of PCD-CT and developing standardized protocols for its application as a paleontological tool of discovery.

## Figures and Tables

**Table 1:** Weights and dimensions of dinosaur fossils.

	Dentary of *Tyrannosaurus*	Skull of *Camarasaurus*
Weight (kg)	10	80 *
Length (cm)	89	105
Depth (cm)	28	85 (including lower jaws)
Width (cm)	10	25 (including plaster and wood base)

* The sample was placed on a plaster base and was too large to be accurately weighed, but it weighed approximately 80 kg.

**Table 2:** Experimental details for dinosaur CT scans.

	*Tyrannosaurus*		*Camarasaurus*	
Parameters	PCD-CT	EID-CT	PCD-CT	EID-CT
Slice thickness (mm)	0.2	0.5	0.2	0.5
Slice interval (mm)	0.15	0.3	0.15	0.3
Rows	120	80	120	80
Rotation time (sec)	1	0.75	1	1
Helical pitch	0.8	0.813	0.8	0.813
kVp setting	140	120	140	120
CTDI vol (mGy)	9.06	6.3	36.4	19.4
DLP (mGy/cm)	851	624.9	4509	2461.9
Focus size	S	L	S	L
Output image	70 keV	120 kVp	70 keV	120 kVp
Matrix size	1024 × 1024	512 × 512	1024 × 1024	512 × 512
FOV (mm)	320	320	500	500
Image Reconstruction	QIR *	AiCE ^#^	QIR *	AiCE ^#^

* Quantum iterative reconstruction (strength level, 3) and ^#^ Advanced intelligent Clear-IQ Engine (deep learning reconstruction).

## Data Availability

The files/data used to support the findings of this study are available from the corresponding author upon request.
